# Active Actions in the Extraction of Urban Objects for Information Quality and Knowledge Recommendation with Machine Learning

**DOI:** 10.3390/s23010138

**Published:** 2022-12-23

**Authors:** Luis Augusto Silva, André Sales Mendes, Héctor Sánchez San Blas, Lia Caetano Bastos, Alexandre Leopoldo Gonçalves, André Fabiano de Moraes

**Affiliations:** 1 Expert Systems and Applications Lab (ESALAB), Faculty of Science, University of Salamanca, 37008 Salamanca, Spain; 2 Department of Knowledge Engineering and Management, Federal University of Santa Catarina, Florianopolis 88040-900, Brazil; 3 Department Information Technology, IT Institute Federal of Science Technology IFC, Camboriú 88340-055, Brazil

**Keywords:** machine learning, information extraction, object spatial, smart cities, gis detection

## Abstract

Due to the increasing urban development, it has become important for municipalities to permanently understand land use and ecological processes, and make cities smart and sustainable by implementing technological tools for land monitoring. An important problem is the absence of technologies that certify the quality of information for the creation of strategies. In this context, expressive volumes of data are used, requiring great effort to understand their structures, and then access information with the desired quality. This study are designed to provide an initial response to the need for mapping zones in the city of Itajaí (SC), Brazil. The solution proposes to aid object recognition employing object-based classifiers OneR, NaiveBayes, J48, IBk, and Hoeffding Tree algorithms used together with GeoDMA, and a first approach in the use of Region-based Convolutional Neural Network (R-CNN) and the YOLO algorithm. All this is to characterize vegetation zones, exposed soil zones, asphalt, and buildings within an urban and rural area. Through the implemented model for active identification of geospatial objects with similarity levels, it was possible to apply the data crossover after detecting the best classifier with accuracy (85%) and the kappa agreement coefficient (76%). The case study presents the dynamics of urban and rural expansion, where expressive volumes of data are obtained and submitted to different methods of cataloging and preparation to subsidize rapid control actions. Finally, the research describes a practical and systematic approach, evaluating the extraction of information to the recommendation of knowledge with greater scientific relevance. Allowing the methods presented to apply the calibration of values for each object, to achieve results with greater accuracy, which is proposed to help improve conservation and management decisions related to the zones within the city, leaving as a legacy the construction of a minimum technological infrastructure to support the decision.

## 1. Introduction

Constant changes occur due to technological evolution in various areas of knowledge, generating new evolutionary cycles, bringing demands, and presenting a large number of still fragmented solutions. For example, the laws of fiscal responsibility, access to information, the civil mark of the internet, and the general law of data protection, guarantee the right to be well informed about what is produced in the public sector. They regulate the implementation of general concepts on data protection, rules for active transparency, and operational procedures. However, the provision of public data in an open format aims to ensure the transparency of stored information that is not under secrecy or access restriction to strengthen the quality of the services offered.

There has been less previous evidence in some studies from the context, focused on analyzing the evolution of data quality [1], basic probability [2], information quality evaluation method [3], text mining techniques [4], research on data and information quality [5], evaluation methods for information quality criteria [6], and mainly higher accuracy for data quality [7]. Innovative and important aspects are highlighted for the main models applied in object learning, and are still being adopted in conjunction with numerous solutions and methodologies. However, different forms and implementation strategies were observed, and among these strategies that enable the monitoring of urban and rural areas, it is essentially necessary to collect and update large volumes of data, consequently of the improvement in the quality of information for the delineation of territorial and social expansion policies.

Until a few years ago, the processes of cartographic revision and, particularly, those aimed at calculating the fiscal area have always been carried out manually. Specifically, these processes required large investments in airplanes or helicopters, making the processes more expensive. Because of this, municipalities are unable to perform mapping surveys frequently. Currently, one of the most current fields of research is to investigate technological capabilities for local authorities to perform detailed surveys of the territory of municipalities at a reasonable cost.

Remote sensing studies based on very high-resolution images have increased in the last few years (e.g., [8,9,10,11,12]), partly because of the availability of satellite images worldwide and the popularization of unmanned aerial vehicles (UAV). The images acquired in all these processes differ in scale, resolution, sensor type, orientation, quality, and ambient illumination conditions. In addition to these difficulties, buildings, cities, neighborhoods, rivers and vegetation may have complicated structures and could be hidden by each other. Both structural and deterministic clues must be taken into account when constructing the solution. Up-to-date and accurate data are essential for municipalities, applied at the Smart Cities concept, that use disruptive technology to solve some specifics problems, in this case, to solve this problem, the solution is the use of satellite and UAV imagery [13] in combination with machine learning techniques [14,15].

However, it is necessary to treat large volumes of data with caution [16], adopting computational and technological resources in conjunction with appropriate use of machine learning methods and techniques. This reflection is based on the proposed [17], in which it had identified that machine learning in several cases had lost connection with problems for other issues due to its complexity. From this perspective, limitations are noted in many existing datasets, metrics employed for evaluation, and the degree to which results express the domain of a problem. As [18,19,20] state, changes are needed in the way research is conducted to increase the impact of ML, and six impact challenges are highlighted to focus explicitly on problems. Aiming to inspire further discussions and focus on the implementation of ML is the main contribution of this paper, highlighting: (1) regulatory framework for use and implementation, (2) cost reduction with rules for informed decision making, (3) avoiding conflicts of interest between nations, (4) extending cyber security through defenses, (5) saving human lives with diagnostics or recommendations, and (6) improving the Human Development Index (HDI) with at least 10% fair taxation in the country. In the following, this study describes evaluation of the main recognition and extraction methods for active identification of geospatial objects, their characteristics, processes, relationships, and integration for rule generation.

The contributions of this paper are summarized as follows: (a) acquisition of spatial data and satellite images; (b) image processing and machine learning; (c) use of GeoDMA (Geographic Data Mining Analyst) and TerraView technologies for remote sensing; (d) use of Weka software applied to the spatial and geographic context; and (e) use deep learning techniques for object detection in GIS images and GIS detection.

To be able to make all the contributions, a methodology of standardization of the steps is applied with the DSR (Design Science Research) proposed by [21], to add new practices and build a literature review in parallel to the technological steps that help in the implementation of the proposal. To this end, the processes of (a) classification in the context of remote sensing (RS) are also reviewed; (b) text mining techniques with the software Weka (workbench for machine learning) performing text analysis, quantifying the words and extracting statistics with the TF-IDF method (Frequency-Inverse Document Frequency). Through a case study carried out in the municipality of Itajaí in the state of Santa Catarina, Brazil. The results collected for the urban and regional planning evaluation period are presented, considering the crossing of environmental and social data, referring to territorial occupation.

For a better understanding of the research and results of this work, the paper is organized as follows: Section 2 presents the development; Section 3 describes the methodology, in Section 4 the materials and methods used to apply the case study are detailed; in Section 5 we present the discussion of the results, and finally, in Section 6 are the final conclusions and future work.

## 2. Development

To develop the project, actions were initially planned for the acquisition of spatial data and satellite images. In the second stage, cataloging and standardization processes are carried out for data interoperability. The third stage presents the technological solutions adopted from GeoDMA and TerraView for remote sensing (RS). In the fourth step occurs the Weka implementation applied to the spatial context. To orchestrate the processes, the main methodology of the work aims from the experiments to define the best practices for the classification process focused on the SR, enabling the integration of all processes towards the active learning of objects. At the end, the case study is presented with specific results and discussions.

### 2.1. Spatial Data Acquisition and Satellite Imagery

In particular for matrix data, [22] defines spatial data acquisition from images from a few approaches, those being: input data definition, segmentation, detection cycles, cell space (matrix) creation, and preliminary extraction features. From the proposals [23,24,25] implemented through the GeoDMA framework (GEOBIA), in synthesis provides the realization of the steps of segmentation of satellite images, extraction of attributes, creation of classification rules, hierarchical classification and visualization of results. Additionally, the works [19,26,27,28,29] describe in detail the precautions to be taken in image acquisition and processing. In particular, according to [27,30], the monitoring of the interactions with the terrestrial surfaces is very important, where each intensity of the solar radiation must be observed. That is, the intensity of solar radiation received by the surface depends on the solar zenith angle that is calculated, considering [27,31] the incident solar radiation, the solar radiation intensity and the solar zenith angle. After acquiring the digital data from the sources provided according to [32], new processes for cataloging emerge. However, the extraction characteristics, initially present the need for specific module of resources extraction and storage in a database. From this, it is possible to extract several features, depending on the size of the scanning process and the amount of objects this task can be very time consuming to complete. Therefore, cataloging guarantees that the features will be extracted without losing original characteristics. Experiments conducted using the images collected by the CBERS-4 (China-Brazil Earth Resources Satellite) and the CBERS-4A satellite, located in http://www.dgi.inpe.br/documentacao/dgi/documentacao/satelites/cbers/capa-cbers (accessed on 1 June 2021), considering different periods.

### 2.2. Machine Learning and Processing

The availability of images from satellites and aerial platforms over the Earth’s surface in the most diverse resolutions has been enabling an unprecedented approach between technology and society, as [28] the processing of large volumes of data and geolocation for the use of mobile devices in most different devices makes the insertion of various technologies flexible. However, large volumes of data are generated, and for analysis, new challenges arise involving interoperability, from those related to data collection and storage, through ethics and privacy [33,34,35], to the development of efficient and robust algorithms to extract the most unimaginable information.

However, processing large volumes of data requires technical expertise in remote sensing, raw data processing, information extraction, a transformation of orthogonal models and spectral mixing, calculation of physical indices, arithmetic operations, frequencies, and statistical classification of data. However, all of these resources seek to assist in classifying image pixels associated with the presence of observed spatial object characteristics. To enable this, only with the use of consolidated technologies as [36,37], various classification algorithms have been developed, as there is a growth in the data obtained by images.

**Figure 1:**
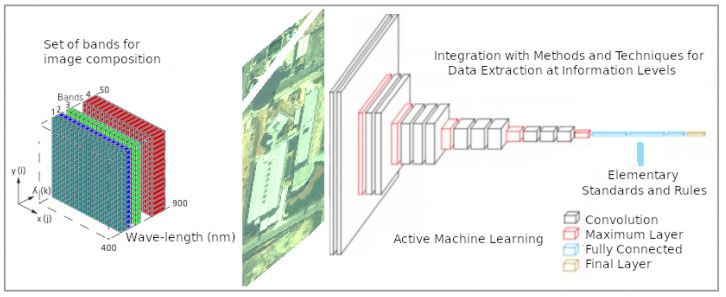
Architecture of the ecosystem adopted for active detection, extraction, and learning of geospatial objects.

Each classifier has its strengths and weaknesses. Hybridizing classifiers with each other have the potential to combine the strengths and overcome the weaknesses by analyzing level by level as per Figure 1.

It is necessary to advance with new studies on the behavior of different algorithms for hybrid classification as KNN (K-Nearest Neighbors) and SVM (Support Vector Machine) addressed by [38], with genetic programming, decision trees with an artificial neural network, Naive Bayes with decision trees and decision trees with K-means. Additionally, in hybrid image processing, specifically for the segmentation process, basic procedures are established according to the works of [22,39], and there are still procedures to be evaluated.

For the processing of images according to [29], together with the extraction of attributes from the regions and their spectral characteristics, they must be previously identified, validated, and calculated. The attributes can be determinant in interpretations and classification processes that involve many classes and some with little separability.

From this, the challenge arises to increase the amount of coherent information to facilitate the discrimination of spectrally similar classes. To this end, determining procedures that help increase identification with greater accuracy provides a set of rules that can be instrumental in identifying distributed objects.

For image processing according to [29], besides the extraction of attributes from the regions and their spectral characteristics, they must be previously identified, validated and calculated. The attributes can be determinant in interpretations and classification processes that involve many classes and some with little separability. From this, the challenge arises to increase the amount of coherent information to facilitate the discrimination of spectrally similar classes.

To this end, determining procedures that help increase identification with greater accuracy provides a set of rules that can be instrumental in identifying distributed objects. However, new attributes can be associated with the spectral attributes from new dimensions attached to the original image. The spectral attributes refer to the color of the pixels and are calculated separately for each band of the input image based on the pixels belonging to the segment.

As per categorical data analysis [40], three standards of texture verification are still evaluated: structural, statistical, and spectral approaches. A statistical approach is the most widely used and considers the texture of an image as a quantitative measure of the arrangement of intensities in a given region. In this context, the concurrency matrix contemplates the numerical characteristics of the texture using similar shades of gray between a pixel and its adjacencies determined by the *N* × *N* pixel quadrant. The main formulations adopted to quantify the texture in digital images, in this case, obtained by (the CBERS-4 satellite), are mean, variance and entropy. The mean, according to (1), corresponds to the value of the arithmetic mean of the gray levels of a region in each band of the image. Where, *R*(*i*) equals each element (*i*) segment *R* and *N* the total number of pixels.

(1) $$Average=\left(\sum_{i=1}^{N}.Ri\right)N$$

(2) $$Variance=\left(\sum_{i=1}^{N}.i-M^{2}\right)$$

(3) $$Entropy=\left(\sum_{i=1}^{N}.Pi.InPI\right)$$

The variance (2) is a measure of the dispersion of the gray level values of the pixels in the region around the mean, and M is the mean of the gray levels of the segment. Entropy (3) is calculated based on the distribution of pixel values in the region and is a measure equivalent to the “distortion” of the values in the region. Where *P*(*i*) contains a normalized histogram of the segment elements. The geometric attributes [29] are calculated based on the polygon that defines the segment boundary, being: area, perimeter, compactness, convexity, and elongation. In other words, the geometric metrics of a segment are defined in advance for the behavior of the processing algorithm. The main considerations about image segmentation refer to the selection of the optimal parameters for each application. However, new active and adaptive processes have presented important results with machine learning, such as GeoDMA.

### 2.3. GeoDMA and TerraVIEW for Remote Sensing

To analyze altered patterns [22,23], in large remote sensing datasets, GeoDMA was created. Implemented in TerraView software, a tool to integrate the most essential image analysis algorithms, ecology metrics, a scheme for multitemporal analysis [41] and data mining techniques to automate the analysis of large databases. Addressing only implementation aspects of active extraction features, it seeks to provide new perspectives for generating automaton functions for data collection, management, analysis, and representation, both for basic functionalities, and the integration extraction, and transformation of geospatial objects [19].

### 2.4. Weka Applied to the Spatial and Geographic Context

Through the work environment for machine learning Weka, it is possible to perform various analyses on a specific data set, or on several sets, provided that these sets have the format in which it can perform the analyses. In this regard, several discussions about GDPM (Geographic Data Preprocessing Module) [23,42,43] arise, extending the Weka Data Mining Toolkit to support geographic data. Additionally, [22] presents discussions of geographic data integration techniques, for example, the ID3, C4.5, and C5.0 algorithms for rule generation. Improvements with Weka-3.9.3 (2019), operating through MOA (Massive Online Analysis) different types of datasets available at http://moa.cms.waikato.ac.nz/downloads (accessed on 20 January 2022).

That is, types such as JSON, XML, SHP, DAT, TXT, CSV, PostgreSQL/PostGIS tables, MySQL/MyGIS, ARFF and XRRF, among others, must necessarily be evaluated and formatted for machine learning processes. The discretization process of Weka is another interesting way to enhance the processes and information extraction, considering the geospatial representation. However, it is necessary to improve experiments for the automation of many of the data transformation tasks for the generation of information with higher quality [44,45].

### 2.5. Deep Learning and Object Detection

The main problem that arises in the processes of acquiring knowledge from images is that of relating the images collected by satellites or drones to object detection systems and the corresponding verification of the same within the databases of local systems. One approach may be to follow the advances in the machine learning algorithm literature, with a focus on using Deep Learning (DL), which is a class of Machine Learning algorithms. This type of algorithm uses multiple layers to progressively extract features from the input images [46].

DL-based approaches are efficient when large datasets are available. The word deep specifies more layers and deep neural networks. DL uses nonlinear functions. Thanks to deep learning, Intelligent Document Processing (IDP) is able to combine various AI technologies not only to automatically classify photographs, but also to describe the different elements of images. Deep learning models, with their multi-level structures, are very useful for extracting complicated information from input images. Convolutional Neural Networks (CNN) are also able to dramatically reduce computational time by leveraging the GPU for computation, something that many networks do not utilize. In the field of object identification in images, two methods stand out: regional proposal algorithms and regression object detection algorithms.

The first method is to discover in advance the possible target locations to be detected in the picture. This can ensure that the highest retrieval rate is maintained when fewer windows are selected. Suppose an image is input and, after a series of convolutions and backbone clustering, a feature map of size M × N is obtained, which corresponds to the division of the original image into areas M × N. The center of each area of the original image is represented by the coordinates of a pixel in this feature map.

**Figure 2:**
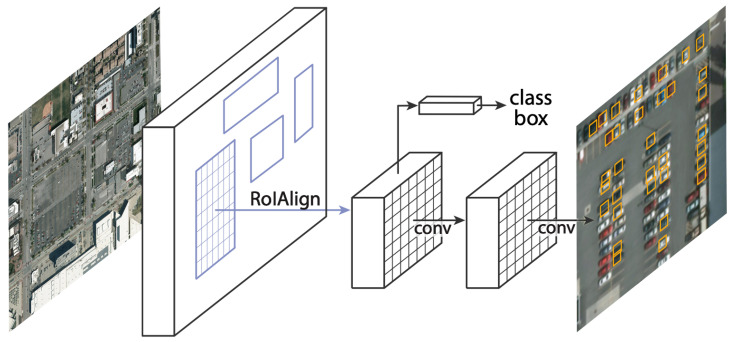
Mask R-CNN framework.

Region Proposition Algorithms are used to determine whether the k anchor boxes corresponding to each pixel contain a target. The network must learn to classify the anchor boxes as background or foreground. From this, it must calculate regression coefficients to modify the position, width and height of the foreground anchor box. Within these classifiers, we find algorithms such as R-CNN [47], Fast R-CNN [48], Faster R-CNN [49] and MASK-CNN [50]. Of the algorithms mentioned above, Mask R-CNN stands out. This algorithm is an extension of Faster R-CNN and works by adding a branch to predict an object mask in parallel with the existing branch for bounding box recognition. The key element of R-CNN Mask is pixel-to-pixel alignment, which is the main missing piece in Fast/Faster R-CNN. The R-CNN mask adopts the same two-phase procedure with an identical first phase (which is RPN). In the second phase, in parallel with class prediction and box clearing, Mask R-CNN also produces a binary mask for each RoI. This is in contrast to more recent systems, where classification depends on mask predictions. Furthermore, Mask R-CNN is simple to implement and train thanks to the faster R-CNN framework, which facilitates a wide range of flexible architecture designs. Furthermore, the mask branch only adds a small computational overhead, allowing for a fast system and rapid experimentation. Figure 2 shows a visual example of the segmentation performed by the algorithm.

**Figure 3:**
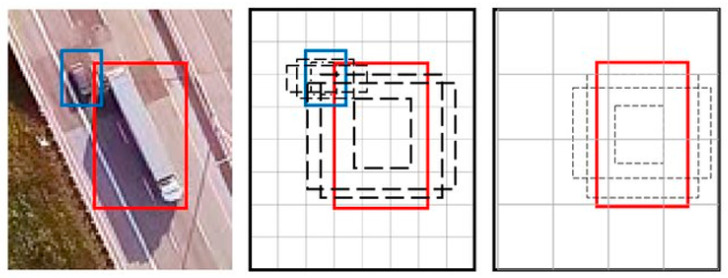
SSD framework.

The above algorithms use detection as a classification problem, i.e., object proposals are first generated and then these proposals are sent to the classification/regression regions. However, some methods approach detection as a regression problem based on a similar operation. Within this field, the YOLO (You Only Look Once) and SSD (Single Shot Detector) algorithms stand out. The SSD algorithm [51] strikes a good balance between speed and accuracy. SSD runs a convolutional network on the input image only once and computes a feature map. It then runs a small 3 × 3 convolutional kernel on this feature map to predict bounding boxes and classification probability. SSD also uses anchor boxes in various aspect ratios, similar to Faster-RCNN, and learns the offset instead of learning the box. To handle scale, SSD predicts bounding boxes after multiple convolutional layers. Since each convolutional layer operates at a different scale, it is able to detect objects of various scales. An example of how the SSD algorithm works can be seen in Figure 3.

**Figure 4:**
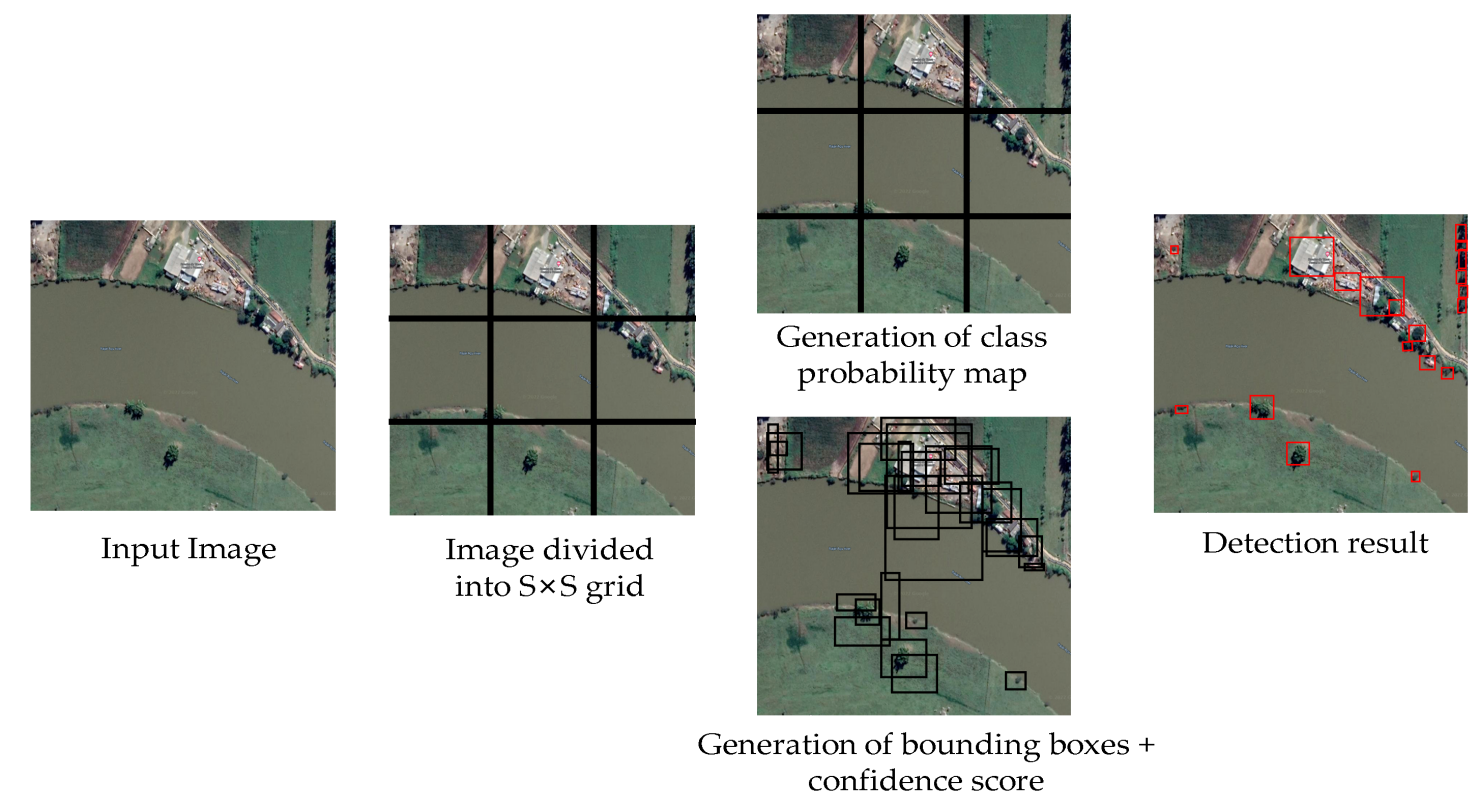
Yolo framework example.

For YOLO [52], detection is a simple regression problem that takes an input image and learns the class probabilities along with the coordinates of the bounding box. YOLO divides each image into an S × S grid, and each grid predicts N bounding boxes along with their confidence. The confidence reflects the accuracy of the bounding box and whether the bounding box actually contains an object, regardless of the class. YOLO also predicts the classification score of each bounding box for each class in the training. It can combine both classes to calculate the probability that each class is present in a predicted box. Thus, a total of SxSxN bounding boxes are predicted. However, most of these boxes have low confidence scores, so if we set a threshold, for example of 30% confidence, we can eliminate most of them, as shown in Figure 4.

YOLO is a faster algorithm than all other detection algorithms, allowing it to be run in real time. Another key difference is that YOLO sees the entire image at once, rather than looking only at the proposals of a region generated in previous methods. Thus, this contextual information helps to avoid false positives. However, one of the limitations of YOLO is that it only predicts one type of class in a grid, so it has difficulties with very small objects. There are several versions of YOLO such as YOLOv2 [53], YOLOv3 [54], YOLOv4 [55], YOLOv4-tiny [56,57], YOLO-Fine [58] and recently YOLOv7 [59]. There are also available versions of YOLO applied to Satellite Imagery, such as YOLT [60] and MRFF-YOLO [61].

## 3. Methodology

From the concepts of DSR (Design Science Research) proposed by [21], whose study considers it essential to also deepen the area of management. In this context, according to [62], hierarchies are applied for knowledge-intensive tasks on each identified problem. Added to the discussions of [24,63,64,65,66] allied to the classification methods being divided according to the processing, into visual or digital, known as supervised, unsupervised and hybrid as per [36].

**Figure 5:**
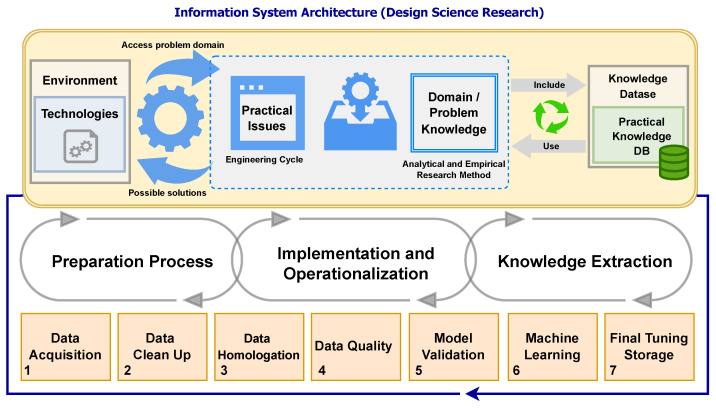
Architecture of the ecosystem adopted for active detection, extraction and learning of geospatial objects.

Additionally, observing the metrics, in parametric or non-parametric and according to the approach by pixel or by regions (objects), the methodology proposed in the work aims, from the survey of satellite images and/or images obtained by RPAS and also by crossing previously shared textual data, to identify through active learning the recognition of geospatial objects with the generation of elementary rules. For this, an architecture for systematic detection and extraction supported by machine learning is proposed, see Figure 5.

In stage 1 meetings, interviews, surveys for questionnaires implementation, documentation for support, and a survey of the prerequisites of the required project are planned. The installation, testing, and homologation processes are also planned. This is where different work platforms are made available for individual or collaborative use (groupware) for integration and standardization. In stage 2, the requirements engineering processes are carried out with the construction of artifacts using UML (Unified Modeling Language). The important delimitation of the coverage area also takes place with the objective of project execution. Data acquisition processes, images, and related documents. The cataloging of data with centralized and shared storage. The processes of treatment, qualification, and homologation of the collected data with due certification. In step 3, acquisitions are made, such as contracting satellite image collection services with specific parameters. Scene processing for example (CBERS) and (LANDSAT-8). Definition of scenes imaged by the satellites through date parameters, bands, and other relevant details, and also the survey and integration of demands with the definition of the goals.

Step 4 is homologation and image processing, with the choice of the contrast method chosen for the visualization of the objects to be evaluated. Optionally, the methods can vary; for example, the linear method, histogram equalization, square contrast, square root contrast, log contrast, mean and standard deviation, decorrelation enhancement, cumulative 2% enhancement, composition and decomposition method, arithmetic operations of image bands with NDBI (Normalized Difference Built-in Index), fusion method, and image segmentation method.

In step 5, the homologation of each processing generated by the choice and application of the methods is subsequently performed, the indexes are prepared, and the methods for detecting and extracting spatial or textual objects are made specifically available. The data structures generated in the previous step are necessarily reused, according to cataloging by date, time, function, data sources, and coverage regions, enabling the import and centralized integration for sharing, through specific infrastructure for networks and sensors.

In step 6, specifically different algorithms for adaptive rule generation are evaluated. Adaptive rules are statistical patterns detected for representing the analyzed dataset. This made it possible to combine them with the intersection of new attributes already stored in the repository. In step 7, the optimization of the data structures for the repository and subsequent reverse engineering is a priority. From known rules, it is possible to actively generate learning about the experiences with the availability of large volumes of data to support the other decision-support processes. To synchronize each step and process, an infrastructure [67,68,69] computer network for remote communication between various devices, data collectors, cameras, and sensors is implemented.

**Figure 6:**
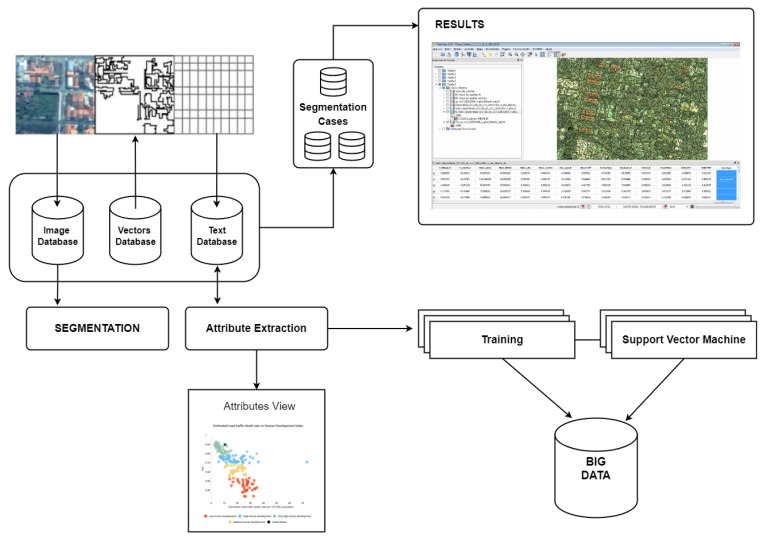
Adapted architecture for systematization and generation of recommendation cases.

Furthermore, through the fruit of several research discussions comes the development of the model for active knowledge extraction, presented by [45], intensifying the interoperability of the data and the advancement of the implementation of the concepts in this work. It also aims at improving the functions through the prototype as presented in Figure 6. From the application architecture idealized by [22], the same also provides the opportunity for the derivation of new experiments for machine learning, since the collection of data, cataloging, discretization of data and application of algorithms is of great importance for the detailing of each process and recording of operations for possible control.

This defines a process for detecting geographic patterns, urban and rural devices through segmentation and other records for automatic observation and evaluation of territorial expansion from the extraction of shared knowledge.

One of the main reasons is the difficulty of constantly processing a large volume of data, due to satellite images collected periodically and which can present files with expressive sizes (megabytes or gigabytes), fundamentally important for pattern recognition.

### 3.1. Remote Sensing Classification

In applications that require image classification, the availability of labeled samples (training data) is closely associated with the choice the analyst will make for extracting information from the images. Two families of techniques are distinguished, called supervised and unsupervised, according to the presence or absence of labeled samples, respectively.

In the context of SR, classification is the process that seeks to assign a label to certain data described by a set of attributes. In digital terrestrial remote sensing imagery, this process is equivalent to determining, for each pixel, which category is present on the surface, such as water, soil, and forest, which is usually done by spectral attributes, such as the gray level in each band.

They are commonly used over the radiometric indices as [70], arithmetic contrast operations with NDBI, NDVI (Normalized Difference Vegetation Index), and NDWI (Normalized Difference Water Index). Considering the processes of unsupervised classification in (SR) and supervised classification, from data collected by (satellites) or unmanned aircraft, these images can be analyzed in different scenarios, whether knowledgeable or not about the observed area. The discussions of [29] describe in detail the implementation of each process that can be adapted to different experiments.

### 3.2. Active Training and Machine Learning

We also consider using parametric classifiers that model the decision boundaries between training classes with a fixed number of parameters, regardless of the number of samples [71,72].

It is the simplest classifier in existence and therefore ends up having a more didactic than operational role. The decision boundaries are positioned on lines equidistant between midpoints of the various classes present.

**Figure 7:**
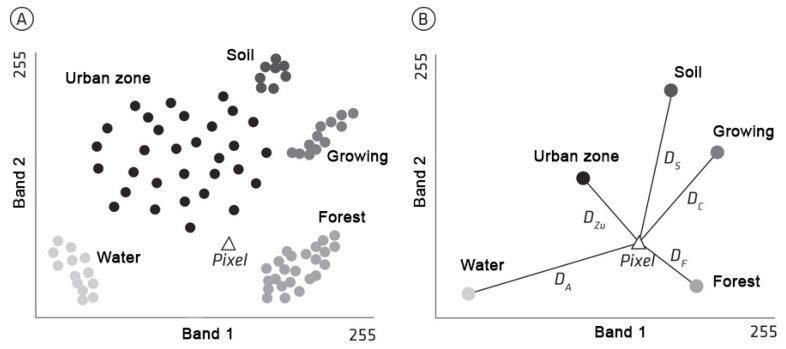
Representation of Euclidean distances.

The classification process by Euclidean minimum distance is performed by Figure 7A the distribution of the sample elements of each class in two bands of a generic image and Figure 7B the averages calculated for each sample and respective distances to a pixel to be classified.

Especially with the evolution of text mining techniques according to [36], the development of the StringToWordVector function can optionally assist in scanning large amounts of text using TF-IDF concepts in Weka treated IDFTransform and TFTransform.

Additionally, when searching with textual data, a statistical measure is adopted that is intended to indicate the importance of a word in a document relative to a collection of documents or in a linguistic corpus. According to [73], it is possible to distinguish the importance between different word features, and it is necessary to calculate the weights of the prominent words. For this, the TF-IDF method is implemented and used to calculate the weight according to (4).

(4) $$TF-IDF=tfxid=\left(\frac{a}{t}\right)Xlog\left(\frac{b}{c+1}\right)$$

**Figure 8:**
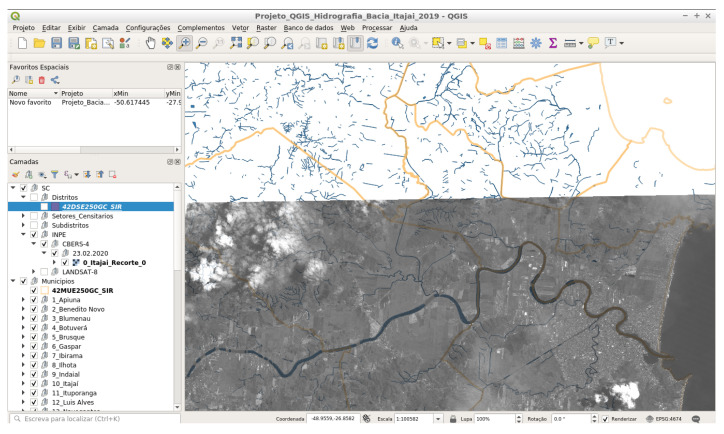
Integration in QGIS with CBERS4 satellite images and CTM data (Itajaí-SC) for monitoring the expansion on riverbanks.

In the formula, *a* is the frequency of the resource in the document set, this is the total number of times of all resources in the document set, *b* is the document number in the document set, and *t* is the number of documents that contain the resource. Then, with the use of the TF-IDF method, it is possible to select n resources with the maximum value of TF-IDF as per the candidate resource set. Using IDFTransform and TFTransform, scans and learning are performed on the datasets prepared for the textual data matching, as detailed in the case study of Figure 8.

This enabled the cataloging of each geographic object properly identified from the satellite image with the items found with IDF-TFTranform. In parallel, from the discussions of [74], another viable strategy to relate and spatially represent different information can be through a geographical matrix of spatial queries, being a two-dimensional representation of intrinsic relationships between locations. To exemplify, implement the forest code and limits of permanent preservation areas in each municipality. Many impasses arise, and although this is not a complex task, it requires great human effort and skilled labor for permanent monitoring. In this sense, the municipalities that make up the basin of the Itajaí-Açú River were mapped to generate the geographical matrix. Through the implementation, the integration of data was carried out, resulting in a large volume of distinct information, providing important relationships for the evolution of monitoring through the recognition of spatial objects as explored in the case study.

## 4. Materials and Methods

### 4.1. Study Area

**Figure 9:**
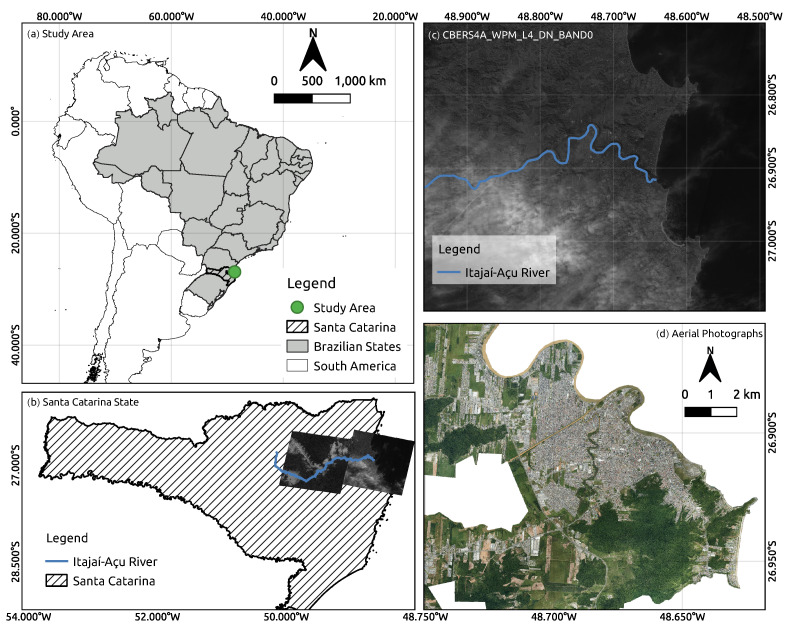
Location of study sites: (**a**) in South America, Brazil, Santa Catarina state. (**b**) State of Santa Catarina with the CBERS4A Panchromatic 2 m Image, including the Itajaí-Açu River layer. (**c**) Satellite image from the CBERS4A of the mouth of the Itajaí-Açu River (**d**) Airborne from the Itajaí—SC Municipality.

The study sites were located in the south of Brazil, in the Itajaí Municipality along the Itajaí-Açu basin river, in Santa Catarina (SC) state, Brazil (Figure 9).

### 4.2. Materials

New challenges arise with updating a municipality’s land registry. According to [73], a good cadastre contributes to the equitable distribution of tax resources, promotes property security and creates bases for urban and regional planning. For this, bases for urban and regional planning are created, using the Multifinality Technical Cadastre (*Cadastro Técnico Multifinalitário*—CTM), available at https://geoitajai.github.io/geo/plantacadastral.html (accessed on 20 October 2022). And from the collection of images through the CBERS-4 and CBERS-4A satellite with spatial resolution of 5 and 2 m, respectively http://www.dgi.inpe.br/catalogo (accessed on 20 October 2022). Additionally, the aerial photogrammetry are obtained from the portal https://arcgis.itajai.sc.gov.br/geoitajai/plantacadastral/plantacadastral.html (accessed on 20 October 2022), they are used to identify spatial objects in the municipality of Itajaí, state of Santa Catarina—Brazil. The map layouts were generated using QGIS software [75].

### 4.3. Methods

With the advancement of technologies and the emergence of large volumes of data, it is possible to cross data through automated processes and solutions with greater reliability and precision. The policies are evaluated with the crossing of environmental and social data, referring to territorial occupation, and considering traditional economic, physical, and legal aspects, among others.

However, one of the limiting factors for updating base registers is still the high cost of developing the entire cartographic framework. However, with the advancement of collaborative technologies, more affordable alternatives should be considered, especially for small and medium-sized cities. In this sense, we highlight the possibility of using photogrammetric techniques, since the logistics involved in the operation of these systems are more flexible and economical when compared to cartography by topographic or geodetic techniques, or even conventional photogrammetry with UAV [76].

**Figure 10:**
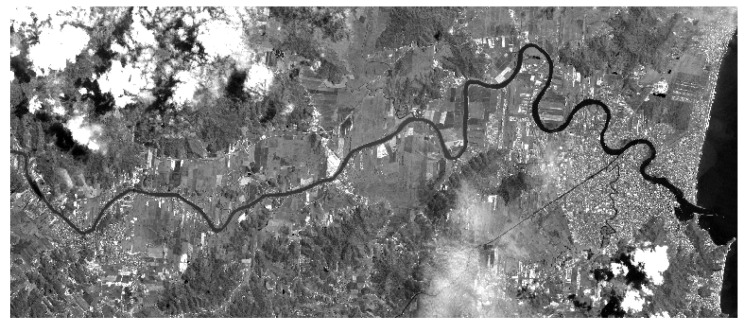
Image of the municipality of Itajaí SC—Brazil, after the contrast method in the area chosen for object detection and active learning.

In the case study, image acquisition processes were carried out, and subsequently the prior selection of the delimited perimeter for application of the present study. Figure 10 presents the result of this process and the application of the linear contrast method. During the investigation, the pixel transformation functions were evaluated from the image contrast, and in the application of active object learning, the contrast presented a more suitable visual result. From the transformation function (T) for a single pixel (r = original pixel value), the resulting pixel value (S) is generated through different techniques to obtain better processing and visualization of objects. Where, (s) = T(r). Table 1 shows the results with the contrast method.

The lower the degree of similarity threshold, the higher the generation of objects for analysis processing and active learning. In other words, higher demand and availability of hardware resources to support higher processing volumes are essential.

Additionally, the availability of a relational database management system, such as PostgreSQL/PostGIS, is another important implementation due to the need of storage for manipulation. The case study was designed to integrate public data with the main objective of evaluating the existence of relationships, direct or indirect crossover. Different data sources were considered for experiments of the algorithms.

After two comparative implementations, over the data set (49,325 occurrences) obtained from the CTM. Where, initially in the first implementation was prioritized application of the classification method to elaborate the decision tree with ID3 algorithm, using the Weka tool (version 2). It generated 78.83% (38,885) correctly classified instances and 21.15% (10,436) incorrectly classified instances.

In the second implementation on the same dataset, a new tree structure was generated with J48 algorithm using Weka tool (version 3.9.3). After visualizing the tree, it was possible to detect the levels and the class (Conservation), with more information gain. For correctly classified instances, it was obtained 85.2002% (42025) of success and 14.79% (7300) not classified. The final kappa statistic of 76.11% also determined the classifier that obtained the best learning. After the implementations with the software Weka, for the same area investigated, another study was performed using the software TerraView/GeoDMA, where the methods of segmentation, image vectorization and later extraction of the set of attributes were applied, see Figure 10.

Subsequently, with GeoDMA for object classification, 212 attributes were generated. From the identification of the characteristics of each pixel was performed the conference of the generation of each polygon and definition of relations for characterization of basic rules of each object. For a better understanding of the objects detected in the study, it was important to evaluate the different types of scales.

## 5. Discussion

From the expected results are presented some reflections and discussions about the development of the work, initially idealized and later obtained. That is, the results achieved by the research and their implementation are presented.

**Reflection 1:** Through the studies presented, was it possible to detect the applicability of knowledge extraction in CTM in conjunction with other areas? Clearly, and as is proven through the case study presented in Figure 5 and Figure 6, along with a comparison of the Weka software in version 3.2 (2001) and version 3.9.3 (2019), both versions allowed for obtaining models, enabling machine learning, and expansion of analysis with new processes of data integration and information extraction.

**Reflection 2:** Specifically in this paper, new performance tests were presented with the Weka classifiers: OneR, IBk, NaiveBayes, and J48. All classifiers use the same CTM data and under the same conditions. That is, at this point it is worth mentioning the inclusion of analysis with the Weka “Hoeffding Tree” classifier, allowing the generation of a tree with less criticality.

**Reflection 3:** Was it possible to use new satellite images and run GeoDMA for automatic learning of new objects? Yes, in this regard, it is worth highlighting the specifications adopted according to Table 1, enabling the correct acquisition of images through standards for the next stage of segmentation, then the vectorization of objects. In particular, several prodecures are performed for image segmentation, which not part of the scope of this work, but will be detailed with new experiments.

**Reflection 4:** Did the development and discussion of the experiments occur with other CTM databases integrated for the recommendation? Partially, some experiments use static datasets successfully, but have not been evaluated by mining continuous stream data using Weka-MOA.

**Reflection 5:** Were performance evaluations of the Weka software performed? Yes, exhaustive performance experiments were conducted, as shown in Table 2 and made available for online access. In all classifiers, the CTM dataset was analyzed with the same parametrizations and specifications, such as the 10-fold cross-validation over the “property conservation” class. The Weka classifiers performed well on a dataset containing 18 attributes and 49,325 instances. The OneR, NaiveBayes, J48, IBk, and Hoeffding Tree classifiers showed satisfactory results. The results of the classifiers are explained one by one below:

For Weka → OneR: OneR processing took 0.09 s to build and run the model. Additionally, with the application of OneR, a correct classification of the instances of (38,404) records was obtained totaling an accuracy of 77.8591% by the algorithm. It also presented satisfactory learning with 63.75% evaluated by the kappa statistic.

For Weka → NaiveBayes: Initially, the processing took 0.13 s to build and run the model. Additionally, with the application of NaiveBayes, a correct classification of the instances of (37,445) records was obtained, totaling an accuracy of 75.9149% by the algorithm. It also presented satisfactory learning with 62.48% evaluated by the kappa statistic.

For Weka → J48: The processing took 2.45 s to build and run the model. Additionally, with an application of J48, a correct classification of the instances of (42,025) records was obtained, totaling an accuracy of 85.2002% by the algorithm. It also showed satisfactory learning with 76.11% evaluated by the kappa statistic.

For Weka → IBk: Processing took 3 min 34 s to build and run the model. Additionally, with an application of IBk, a correct classification of the instances of (40,042) records was obtained, totaling an accuracy of 81.1799% by the algorithm. It also showed satisfactory learning with 69.97% evaluated by the kappa statistic.

For Weka → Hoeffding Tree: Processing took 0.69 s to build and run the model. Additionally, with an application of the Hoeffding Tree, a correct classification of the instances of (39,203) records was obtained, totaling an accuracy of 79.48% by the algorithm. It also presented satisfactory learning with 66.54% evaluated by the kappa statistic.

Experiments with Weka → IBk from the vector with 25 attributes and containing (1,000,000) instances, generated after image segmentation Weka with the IBk classifier built the classification model quickly, but presented a very large slowness (9 h) to measure the distances of all instances. However, the alternative found to speed up the processing was to retain in memory only a “window” of instances, instead of the complete dataset. In Weka, the default parameter “window size = 0” allows you to set the maximum number of instances allowed in the training pool, and adding additional instances simply removes the old ones, freeing up memory to improve performance.

In addition, for a better understanding, analysis was performed on the datasets below, being separated into three different sets to initially compare the training with 100 (objects), 1000 (objects), and 15,000 respective training results, it was possible to decide which algorithm to use first to be prioritized and adopted for further processing, as shown in Table 2, containing the results obtained from training the different datasets and compared with the performance of processing the entire set of objects. The final test was evaluated with a total of 49,325 instances, and these instances are isolated from the training dataset.

With Weka → Hoeffding Tree, a Hoeffding tree (VFDT—Very Fast Decision Trees) is a very fast decision tree algorithm for incremental decision tree induction at any time, capable of learning from massive data streams, assuming the distribution generation instances do not change over time. Hoeffding trees exploit the fact that a small sample can be sufficient to choose an optimal splitting attribute. This idea is supported mathematically by the Hoeffding limit, which quantifies the number of observations (examples) needed to estimate some statistics within a prescribed precision (according to the goodness of an attribute). A theoretically attractive feature of Hoeffding Trees not shared by other additional decision tree learners is that it has good performance guarantees. Using the Hoeffding boundary, one can show that its output is asymptotically nearly identical to that of a non-incremental study using infinite examples proposed by [41]. This classifier is a successful reference in dealing with large spatial representation datasets, for example, the evaluated dataset (Weka→ ConvtypNom), regarding spatial coverage of forests with quadrants defined in 30 × 30 m, 581,012 instances, and 54 attributes, elaborated the model in 41 s and completed the evaluation processing with cross-validation 10 times in 6 min and 49 s. All the results of Weka 3.9.3 performance are presented in Table 3.

In all classifiers, the CTM dataset was analyzed with equal parameterizations and with the same specifications, such as the “k = 10 cross-validation” on the “property conservation” class. For property conservation in the “good condition” category, from the confusion matrix generated by the first classifier established, the true positives (TP) with 22,279 units, and the true negatives (VN) with 19,746, totaling 42,025 units to be certified, were first identified. Afterward, the false positives (FP) with 4002 units enabled the separation for re-evaluation of each occurrence. However, unlike the IBk classifier, the other evaluated classifiers had good performance regarding processing time using 18 attributes and 49,325 instances, over the same computational infrastructure provided.

With the satisfactory results obtained with OneR, NaiveBayes, J48, IBk, and Hoeffding Tree, allows the use of GeoDMA for automatic learning of new objects to be positively proven by Figure 10. Additionally, the fusion between textual classifiers and geospatial classifiers made possible through this work, the verification of an innovative form of knowledge extraction engineering.

From the use of GeoGMA [22], to perform the extraction of attributes, after exhaustive performance tests on the hardware used Table 1, adjustments were applied opting for the selection of all statistical methods, except “Percent of each class by area”, because this method increases the consumption of processing and memory. Still in the process of extraction of attributes, it was possible from this procedure to obtain a better response time with a duration of up to 15 min of processing load.

**Figure 11:**
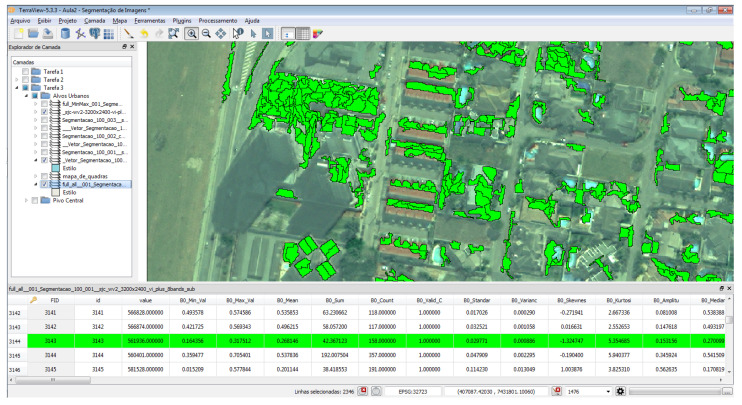
Results after calibration of values and recognition of objects.

Specifically, in Figure 11, it is shown how this made it possible to start the elaboration of queries through the filter on the attribute “B0Mean” > 0.4, especially to obtain the selection of objects with the highest “vegetation index” in the image.

In the second query, it was possible through the filter on the attribute “B7Mean” > 300.0, to obtain the selection of objects with the “shadow” characterization on the image. In this particular case, the query changed the return color for the objects. However, it was not rendered after processing, changing the color parameter set as “Yellow” to “Green”, but remaining the best identification of the color “yellow” for the recognition of the object “shadow” in the image.

**Figure 12:**
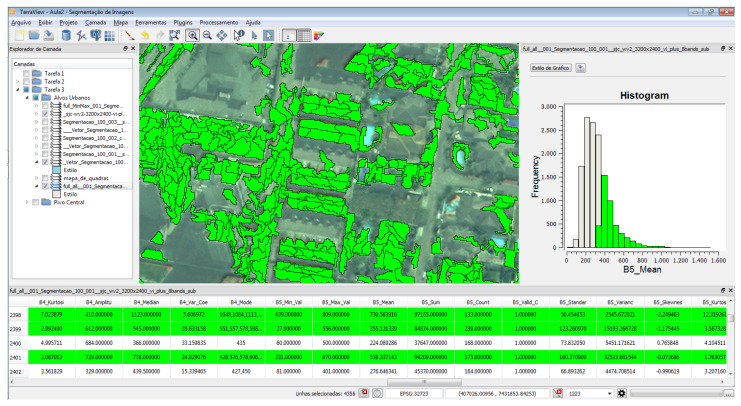
Results after calibration of values and recognition of objects with the results in the historigram.

Still, for the definition and characterization of the objects, some queries were performed, allowing, through the filter on the attribute “Band 5-Mean” using the GeoDMA [22], us to obtain different values for the selection of objects and characterization, such as “ceramic roofs”, as shown in Figure 12. Thus, for the tested objects, the following values were obtained and are available in Table 4.

**Figure 13:**
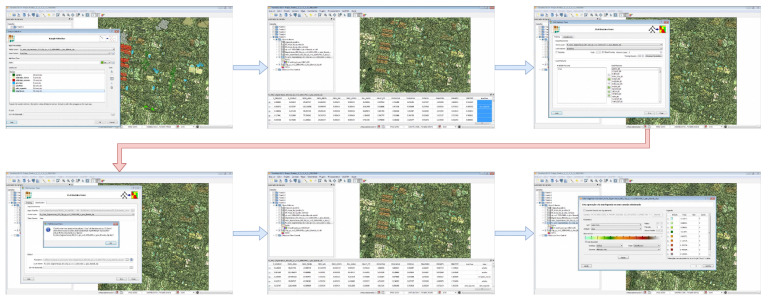
Calibration for segmentation process.

**Figure 14:**
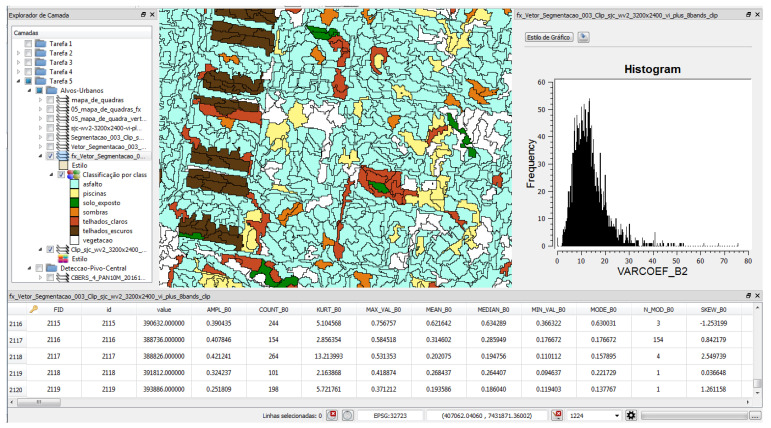
Segmentation results of the recognized objects.

The choice of samples was made randomly within the dataset images used for training, aiming initially to understand how the learning was performed, especially with the use of GeoDMA, as in Figure 13 and Figure 14. This provided an important experience in the choice of classes. Specifically, the classes of investigation were: (a) asphalt, (b) roofs—light, dark or ceramic, (c) swimming pools, (d) shadows, (e) exposed soil, and (f) vegetation. Figure 13 describes the calibration process to obtain the final result, detailed in Figure 14.

From this study, it was possible to carry out new experiments to acquire a larger number of samples and improve the rule generation. For example, with respect to the class “asphalt”, the rule was very broad, in which a greater number of representations were obtained, as illustrated in Figure 14. On the other hand, for “dark ceramic roofs”, the rule was generated with better results. However, for future work, new implementations will be developed, aiming to train new classes.

Certainly, much still has to evolve computationally, especially with regard to constant active machine learning. In particular, urban object recognition and monitoring is noted. All documents related to the Weka training, datasets and other complementary documents are available at http://sadpreaigeo.org/ufsc-egc/mtec2019 and http://sadpreaigeo.org/ufsc-egc/mtec2022 (accessed on 15 December 2022).

## 6. Conclusions

Finally, according to the initial objectives of this work, through extensive research, it was possible to prove the applicability of the extraction of knowledge with the integration of data collected from the Cadastro Técnico Multifinalitário (CTM). Additionally, as prioritized, the research contemplated through investigation of the publications made in the last 5 years. During the study, it was noted that there is a great involvement of the academic and scientific community in the development of technologies that understand the geospatial and earth phenomena.

Growth and strong trends were observed in the use of the SVM (Support Vector Machine) method for evaluating large volumes of textual and geospatial data, as was the use of data discretization to enhance the execution (performance) of classifiers (algorithms). In this sense, the possibility of implementation and integration of the software Weka with TerraView/GeoDMA was proven, and they were compatible and operationalized because both complement each other from the collection to the structuring of textual and geospatial data for the evaluation of datasets, as presented in the Section 5.

An important characteristic to highlight with the Weka software was experimention with vectors that presented only numeric values, in which linear regression (Weka 3.9.3) proved to be faster to deal with large volumes of data. Regarding linear regression, the J48 decision tree showed the best results with the best classifier with accuracy (85%) and the kappa agreement coefficient (76%) in an average time of 0.30 s.

For future work, further studies to advance experiments from evolving data streams—those generated by mechanisms that change or fluctuate over time, by implementing the Weka/MOA package, designed specifically for data stream mining including new adaptations with Deep Learning algorithms. Furthermore, we intend to advance the development of a module for a pre-processing face as proposed [77], prioritizing data collection, transformation, and preparation of datasets and images. This is essential for the crossing of data and construction of rules to ensure the quality of the information to the user and decision maker. Another need concerns the improvement of models, being more or less robust and that can be reusable through vectors for TF-IDF x TerraViewGeoDMA application.

Although great demands of work are generated, one must prioritize care for the quality of data and information both for technical and operational issues, as well as for strategic issues that require constant validations, allowing certifications to occur for each step performed during all the extraction processes, generating a reliability indicator for the quality of information. The continuity of actions to intensify the implementation of processes for information quality, in this work, is indispensable so that all stages of knowledge extraction are guaranteed and certified.

Importantly, our results provide evidence for an implementation of an innovative practical, and systematic approach. The extraction of information and recommendation of knowledge shows a greater scientific relevance. Allowing the methods presented to apply calibration parameters for each object and achieve results with greater accuracy.

## Figures and Tables

**Table 1:** Preparation for active learning experiments.

Satellite	Segmentation			Generated Records
**CBERS** **Sensor** **Banda** ${}^{a}$	**Contrast** **Method** ${}^{b}$	**CPU Time** **i7 6.5 GB** **GNU/Linux**	**Similarity**	**Object** **Detection**
PAN5M—Band Espec = 1	Linear	327 s 70	0.050	1,066,788
		390 s 46	0.045	1,345,492
		435 s 84	0.040	1,639,031
		464 s 94	0.035	2,158,357
		516 s 02	0.030	2,770,752
		572 s 11	0.025	3,760,484

^*a*^ Image acquisition criteria, e.g., (date, location, quality and others). ^*b*^ Software used for image processing, such as TerraLib/TerraView (INPE) and Qgis.

**Table 2:** Training datasets (objects).

Algorithm	Training (100 Objects) %	Training (1000 Objects) %	Training (15,000 Objects) %	Performance Evaluation (Final) %
J48	52	77.9	80.4067	85.2002
IBk	78	78.2	80.4467	81.1799
Hoeffding Tree	76	77.1	80.3733	79.4800
OneR	75	77.9	80.3600	77.8591
NaiveBayes	76	77.1	79.4133	75.9149

Training datasets and evaluation/tests are available in: http://sadpreaigeo.org/ufsc-egc/mtec2022/ (accessed on 15 December 2022).

**Table 3:** Performance comparison of Weka 3.9.3.

Nr	Weka 3.9.3 Classifiers	Time	Correct Instances	% Hits	% Kappa
1${}^{\circ }$	J48	0.30 s	42.0250	85.2002%	76.11%
2${}^{\circ }$	IBk	3 m 34 s	40.0420	81.1799%	69.97%
3${}^{\circ }$	Hoeffding Tree	0.69 s	39.2030	79.4800%	66.54%
4${}^{\circ }$	OneR	0.03 s	38.4040	77.8591%	63.75%
5${}^{\circ }$	NaiveBayes	0.04 s	37.4450	75.9149%	62.48%

Results available in: http://sadpreaigeo.org/ufsc-egc/mtec2019/ (accessed on 20 January 2022).

**Table 4:** Obtained values.

ID	Min	Max	Mean
2128	386.000000	511.000000	426.769231
2135	241.000000	724.000000	425.036585
2240	235.000000	631.000000	532.342541
2302	326.000000	692.000000	531.554622
2398	27.000000	556.000000	355.121339
2999	310.000000	664.000000	551.215962
3056	385.000000	645.000000	553.728000
3075	460.000000	650.000000	596.877160
3116	260.000000	526.000000	426.401042
3144	372.000000	529.000000	436.227848
3732	218.000000	638.000000	425.622951
3767	270.000000	521.000000	391.483974
3768	416.000000	584.000000	529.207207
3867	229.000000	555.000000	398.095023
